# Experience and perceptions of healthcare providers on clinical management and care of near-miss infants: a qualitative content analysis

**DOI:** 10.1186/s12913-023-10097-3

**Published:** 2023-12-13

**Authors:** Mengstu Melkamu Asaye, Kassahun Alemu Gelaye, Yohannes Hailu Matebe, Helena Lindgren, Kerstin Erlandsson

**Affiliations:** 1 Department of Women and Family Health, School of Midwifery, College of Medicine and Health Sciences, University of Gondar, Gondar, Ethiopia; 2 Department of Epidemiology and Biostatistics, Institute of Public Health, College of Medicine and Health Sciences, University of Gondar, Gondar, Ethiopia; 3 Department of Pediatrics and Child Health, School of Medicine, College of Medicine and Health Sciences, University of Gondar, Gondar, Ethiopia; 4 Department of Women’s and Children’s Health, Karolinska Institutet, Solna, Sweden; 5 Sofiahemmet University, Stockholm, Sweden; 6 Department of Women’s and Children’s Health, Solna, Sweden; 7 School of Health and Welfare, Karolinska Institute, Dalarna University, Falun, Sweden

**Keywords:** Experiences, Perceptions, Attitudes, Clinical care, Management, Qualitative content analysis, Near Miss Infants, Midwives, Ethiopia

## Abstract

**Introduction:**

Neonatal Near Miss (NNM) refers to neonates with severe complications who almost died but survived immediately after birth. In Ethiopia, the prevalence of NNM has been assessed using a validated Neonatal Near-Miss Assessment Scale. However, understanding the experiences and perceptions of healthcare providers in the clinical management and care of NNM infants remains unexplored. The aim was to investigate the determinants contributing to the survival of neonatal near-miss babies and to identify any barriers encountered, as reported by the experiences of healthcare providers in public hospitals of Amhara Regional State, northwest Ethiopia.

**Methods:**

Semi structured interviews were used to collect data from 25 midwives, nurses, and pediatricians with at least six months of prior experience in one of the labor wards or neonatal intensive care units at one of the four public health hospitals in the Amhara Regional state of northwest Ethiopia included in a large intervention study assessing a NNM scale. Purposeful sampling was used, selecting participants based on their experiences related to the aim of this study. The participants had a varying level of education and years of experience to care for NNM infants. The average age of the healthcare providers was 31 years, with 7 years of work experience. The transcripts of the interviews with the healthcare providers were analyzed using qualitative content analysis.

**Results:**

The experience and perceptions of healthcare providers was described in the main category “A sense of hopelessness when caring for the baby” capturing a broader emotional and professional aspect, while the subcategories “Unclear responsibilities discharging one’s mission”, “Provision of kangaroo mother care” and “Quick action required at birth” are more specific and practical. Healthcare providers perceived a sense of hopelessness when caring for the NNM infant, particularly providing Kangaroo Mother Care (KMC) and quick actions when required at birth to save the life of the infant.

**Conclusion:**

Unclear responsibilities and a sense of hopelessness could have acted as barriers, hindering the ability of healthcare providers to fulfill their mission of taking swift actions and providing KMC to NNM infants, thus impacting their ability to save the lives of these infants. Healthcare providers’ and parents’ attitudes must be changed towards hope rather than hopelessness when caring for NNM infants.

## Introduction

Neonatal Near Miss (NNM) refers to neonates with severe complications who almost died but survived immediately after birth. In Ethiopia, the prevalence of NNM has been assessed using a validated Neonatal Near-Miss Assessment Scale [1–3]. In the validated Neonatal Near-Miss Assessment Scale [1–3] NNM is defined as neonates who nearly died but survived after birth in hospitals’ labor wards or neonatal intensive care units, meeting at least one criterion of the validated Neonatal Near-Miss Assessment Scale. There are certain medical criteria playing a significant role in Ethiopia [1–3] whereas determinants related to caring also have a causal or influential role in the survival of neonatal near-miss babies in clinical management and care, and these aspects have not been explored or studied yet in Ethiopia. In the validated NNM Assessment Scale [1–3] the medical criteria include pregnancy-induced hypertension, premature rupture of membranes, referral from other health facilities, fetal malposition and prolonged active first stage of labor. To meet the 2030 Sustainable Development Goal (SDG) [4] Ethiopia plans to reduce neonatal mortality from 29 to 12 per 1000 live births by implementing proven and cost-effective newborn survival interventions [5]. Inadequate care and clinical support were in studies from Brazil [6] and Iran [7] found to increase the risk of NNM. Proper resuscitation practices, timely neonatal admission to the Neonatal Intensive Care Unit (NICU), and teamwork among midwives have been identified as essential factors contributing to saving lives. In a systematic review, the results highlight that patient neglect in healthcare institutions could be perceived as a form of disrespect of women, which may lead to a lack of care for newborns [8].

One determinant with the potential to influence the survival of neonatal near-miss babies in clinical care is initiating skin-to-skin contact within one hour of birth to initiate breastfeeding [9, 10]. Midwives, other healthcare providers, and partner support could contribute to survival through exclusive breastfeeding [11], as expressing breast milk can save lives for low birth weight and premature babies [12]. Infants with extremely low birth weight are especially vulnerable to heat loss immediately after birth [13]. The initiation of short intermittent and continuous Kangaroo Mother Care (KMC) therefore significantly reduces the risk of neonatal death [14, 15]. A meta-analysis of RCT studies concluded that KMC is safe and should be encouraged and advocated for [16] when also enhancing mothers’ motivation and bonding with their premature, low birth weight infants [17].

Our research group has used the validated Neonatal Near-Miss Assessment Scale [1, 2] as an audit and feedback system in University of Gondar, Debretabor, and Felegehiwot comprehensive specialized hospitals, as well as Debark General Hospital in the Amhara Regional State of northwest Ethiopia [1–3]. The relationship between fetal malposition, primiparous births, referrals from other health facilities, premature rupture of membranes, and NNM was partially mediated by grade III meconium-stained amniotic fluid and the duration of the active first stage of labor. Early diagnosis of these potential danger signs and appropriate intervention could be of supreme importance in reducing NNM [1–3]. However, determinants that play a causal or influential role in the survival of neonatal near-miss babies in clinical management and care remain unexplored.

Describing healthcare providers’ experiences and perceptions of determinants related to caregiving having a causal or influential role in the survival of neonatal near-miss babies in clinical management and care is crucial for evidence generation and planning interventions in Ethiopian hospitals [5]. By understanding the challenges and successes faced by healthcare providers when caring for NNM babies, we can strive to provide effective and timely care to all women and newborns. To the best of our knowledge, healthcare providers’ experiences and perceptions of caring for NNM babies have not been investigated among healthcare providers involved in clinical management and care in Ethiopian hospitals. With this background, this study aims to investigate the determinants contributing to the survival of neonatal near-miss babies and to identify any barriers encountered, as reported by the experiences of healthcare providers in public hospitals of Amhara Regional State, northwest Ethiopia.

## Method

### Design

A qualitative inductive research design was employed [18], and data were collected through semi-structured individual interviews [19] with healthcare providers representing midwives, nurses, and pediatricians at four public health hospitals in the Amhara Regional State of northwest Ethiopia. Ethical approval was granted by the University of Gondar Institutional Review Board (IRB) (Ref. No: V/P/RCSC/05/2543/2021), and ethical principles were followed in accordance with the Belmont Report [20] and the Helsinki Declaration.

### Setting

The study was conducted at the University of Gondar, Debretabor, and Felegehiwot Comprehensive Specialized Hospitals, as well as Debark General Hospital. In the selected hospitals, there were 380 midwives, 78 neonatal nurses, and 35 pediatricians employed. Each maternity ward was equipped with triage, follow-up, second-stage, and postnatal units. The newborn units were further divided into sections, each staffed with senior doctors, midwives, and nurses. The average monthly birth rate ranged from 135 to 340 [21]. Each year, 2,450 infants were admitted to the neonatal units out of 15,000 deliveries performed in the labor unit. Near-miss neonates who almost died but survived immediately after birth in the labor unit of selected hospitals or within the first day of admission to a neonatal intensive care unit were enrolled in an intervention study in year 2020 based on at least one criterion of the validated Neonatal Near-Miss Assessment Scale [1, 2].

### Study participants

When conducting qualitative studies aimed at obtaining broad descriptions, the recruitment process typically involves a strategy to enlist participants capable of offering diverse perspectives and experiences. Therefore, we employed purposeful sampling, selecting participants based on their experiences related to the aim of this study. The qualitative study participants consisted of 25 midwives, nurses, and pediatricians, each with a minimum of six months’ prior experience in either labor units or neonatal intensive care units at one of the four public health hospitals in the Amhara Regional State of northwest Ethiopia, as included in the previous study [3]. Midwives, nurses, and pediatricians with less than six months’ prior experience in one of the labor wards or neonatal intensive care units at these hospitals were excluded. The average age of the healthcare providers was 31 years, ranging from 27 to 38 years. Of the participants, 72% (18/25) were male, while 28% (7/25) were female. Participants included 36% (9/25) midwives, 36% (9/25) nurses, and 28% (7/25) pediatricians. All midwives and nurses held bachelor’s degrees. Nineteen out of 25 healthcare providers (75%) were married. The average work experience of healthcare providers was 7 years, ranging from 3 to 14 years.

### Interview guide and collection of data

The interview guide was developed in the local language, Amharic. The interview guide was pilot-tested with two healthcare providers, and some modifications, such as changes in the order of questions and the inclusion of more specific and detailed questions, were made.

A letter of permission was issued by the regional health bureau to each hospital administrator. After accepting the invitation, potential participants were provided with oral and written information about the study. Informed consent was obtained, and appointments for individual interviews were scheduled. The included healthcare providers expressed their willingness to communicate and participate in the interviews. Information about the study was provided orally and in writing, and a written consent form was signed by the 25 approached healthcare providers. Semi-structured interviews were conducted by the first author and a note-taker to collect the data. All the interviews were conducted in a private, comfortable office to ensure privacy. The offices were located within their respective hospitals. The process of adding new participants continued until no new content emerged in the interviews.

The interviewer (MMA) posed four main questions to the healthcare providers regarding their experiences and perceptions of clinical management and care influencing the survival of neonatal near-miss infants and uncovering any obstacles encountered in their experiences:

1. Can you describe your experiences with the infants who survived despite initial expectations of them not surviving?
2. In your opinion, what factors made the difference between those who survived and those who did not?
3. What kind of care did you provide that might have made a difference between life and death?
4. What does “NNM” mean to you?

Follow-up questions were added to the main questions:

(a) Could you provide more details about that? (b) What do you mean by that?

The interviews ranged in duration from 30 to 50 min. All interviews were audio-recorded, transcribed, translated into English (as a reference), back-translated, and validated by listening to the audio tape recordings several times. Passwords were employed to secure all study records, and data collection forms were identified using codes. Any record containing a name or other personal identifier was stored separately.

### Data analysis

To ensure the reliability of the results, we carefully analyzed interview transcripts using a step-by-step manual process, following the qualitative content analysis method described by Elo and Kyngas [18]. The research team had previous experience with NNM and with qualitative content analyses, generating codes and subcategories at both a manifest and a more abstract level. Throughout the research, the authors were cautious not to make premature assumptions. We maintained open communication among the authors to encourage thoughtful reflection rather than rushing to conclusions.

We have provided a clear description of our analysis process, enabling replication and instilling trust in our results. The following steps were taken during the data analysis:

1. The interviews were initially read to gain an overall understanding of the content related to the study’s aim and objectives.
2. The interview content was repeatedly read and discussed within the team of reserachers to achieve immersion and a holistic perspective.
3. Each interview text was segmented into meaning units and condensed, extracting contextualized meaning from units longer than a sentence or a shorter paragraph. A contextualized meaning unit captures relevant information for the research’s aim, considering the participants’ perspectives and experiences. Notes and memos taken during the interviews were reviewed to confirm the correctness of the contextualized meaning units; however, these memos were not considered part of the analyzed texts. The text included in the analysis all originated from the transcribed interviews.
4. The contextualized meaning units were labeled with codes that emerged inductively. When all data had been coded it was 48 different codes. A code refers to a label or tag that researchers assign to segments of data during the analysis process.
5. Codes with similar meanings and content were combined and abstracted into subcategories. Subcategories hence consisted of many codes.
6. These subcategories were then related to an overall main category. The content of the main category is a common thread that weaves through all the text synthetically. It is an essential feeling among the healthcare providers when providing quality care to neonatal near-miss infants (as shown in Table 1) [18].

**Table 1:** Qualitative content analysis of transcribed and translated data from interviews with 25 healthcare providers in public hospitals in Amhara Regional State northwest Ethiopia. Example of the analysis process; meaning unit, condensed meaning unit, code, subcategory and category

Meaning unit from the original text	Condensed meaning unit	Code	Subcategory	Main Category
“I recall a 900-gram baby admitted to the hospital at 27 weeks gestation. Because her mother believed she would never grow up, her mother disappeared, leaving the baby in the hospital. We didn’t believe she (the baby) would survive”.	Healthcare providers’ perception was that a child being abandoned and left in the hospital (27 weeks of gestation) would not survive.	Abandoned baby would not survive	Unclear responsibilities discharging one’s mission	A sense of hopelessness when caring for the baby
“I remember a very low birth weight, 35-week-old male baby with hypoglycemia and abnormal vital signs. It was wrapped in cotton, vital signs were taken every two hours, and it was placed in incubator. The mother correctly provided intermittent kangaroo mother care, which contributed to her baby’s survival”.	Kangaroo mother care at neonatal intensive care unit saves lives	Kangaroo mother care saves lives	Provision of kangaroo mother care	
“I quickly cut the cord, wrapped the newborn in a towel and positioned it close to the radiant. I removed the meconium and resuscitated it for 20 min. I reasoned that such actions would assist the baby’s survival”.	Quick action at birth with secretion removal and resuscitation saves lives	Quick action at birth	Quick action required at birth	

## Results

**Fig. 1:**
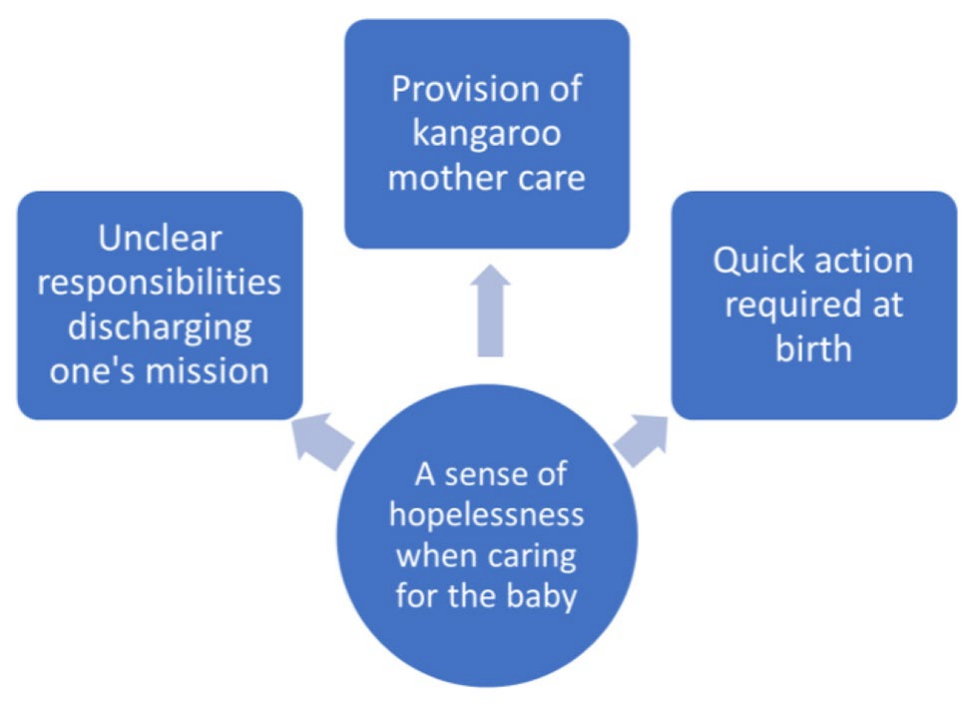
The main category and the three subcategories were revealed through the analysis of interviews with 25 healthcare providers working in public hospitals in the Amhara Regional State of northwest Ethiopia

The experiences and perceptions of healthcare providers regarding the clinical management and care of neonatal near-miss infants were described within the main category “A sense of hopelessness when caring for the baby.“ This main category encapsulates broader emotional and professional aspects, while the subcategories “Unclear responsibilities discharging one’s mission,“ “Provision of kangaroo mother care,“ and “Quick action required at birth” are more specific and practical, directly related to the care and management of neonatal near-miss infants. Healthcare providers perceived a sense of hopelessness when caring for NNM infants, especially when providing KMC and taking quick actions when required at birth to save the life of the infant. The main category and the three subcategories can be found presented in Fig. 1.

### Unclear responsibilities discharging one’s mission

Healthcare providers believed that an infant abandoned and left in the hospital with very low birth weight and an extremely early gestation would not survive. This uncertainty made their responsibilities unclear when it came to managing and caring for the infant. Consequently, these babies would not receive expressed breast milk or maternal care, putting them at risk of infection and other health-related problems, such as low glucose levels. Due to these concerns, healthcare providers doubted the survival chances of such abandoned high-risk infants.

“I recall a 900-gram baby admitted to the hospital at 27 weeks of gestation. Her mother believed she (the baby) would never grow up, so her mother disappeared, leaving the baby in the hospital. We didn’t believe she would survive.“ (Participant no. 6).

Infants with lower estimated chances of survival at birth were often neglected by healthcare providers due to their unclear responsibilities regarding the management and care of NNM infants. Healthcare providers may perceive an infant’s chance of survival as low, especially when dealing with numerous laboring women in the ward. Consequently, they might prioritize other cases and leave laboring mothers alone with fetuses having a low probability of survival at birth. During labor, healthcare providers may perform ultrasound examinations to estimate the expected fetal weight. However, these estimates can sometimes be incorrect or too low.

“I recall a baby girl weighing 1100 grams who was born on the bed alone with her mother. Using ultrasound, the baby’s weight was estimated to be 1200 grams during labor. This baby girl was ignored because healthcare providers had already estimated a lower chance of survival before birth. After birth, the girl was awake and crying when I went to check on her.“ (Participant no. 8)”.

At the neonatal intensive care unit, an infant with a lower survival estimate during the first check-up would often be overlooked by healthcare providers, according to the healthcare providers themselves. Healthcare providers would inform family members about the infant’s condition and its low chance of survival. Consequently, they might limit treatment and care due to the perceived low chance of survival. Parents, however, may exert pressure on healthcare providers to provide more attention, treatment, and care for their baby rather than compromising on management and care. Parents may be fervently hopeful for their baby to survive and may struggle to accept unclear responsibilities among healthcare providers. These unclear responsibilities regarding the management and care of NNM infants led healthcare providers to compromise on treatment and care for high-risk infants.

“The baby was born with a low birth weight and a very early gestational age. We informed the baby’s father that the chance of survival was small, but he refused to believe us. We (healthcare providers) initially felt helpless. He (the baby’s father) asked us, ‘Why are you dampening my hopefulness?‘ He was very eager and urged us to check and treat her (the baby) at every opportunity.“ (Participant no. 22).

Some healthcare providers defined NNM as infants who were close to death but survived due to the care provided. Others perceived the NNM baby as a stillbirth if they did not breathe spontaneously. The unclear responsibilities often led them to neglect NNM babies, especially if they were new to the concept, as healthcare providers explained. Clear responsibilities for each healthcare provider, as per the participants, can be established through in-service training on appropriate treatment and care. Focusing on the healthcare providers’ perceptions of near-miss infants can help ensure appropriate care and treatment. In-service training and raising awareness among healthcare providers can enhance their motivation and satisfaction in caring for such high-risk infants. Participants mentioned that it was frustrating to care for infants with such a low survival rate, such as very premature and low birth weight infants. Their previous experience played a role in providing care and treatment to future high-risk infants.

“Knowing how to properly care for and treat NNM infants provided us with satisfaction, experiences that they may survive, and helped us develop a shared understanding of how to manage a near-miss baby. It taught us a lot on its own.“ (Participant no. 6).

Being familiar with the concept of NNM allowed for special care, strict treatment, and close monitoring of these high-risk babies. Unclear responsibilities regarding how to manage NNM infants sometimes led healthcare providers to neglect this mission, instead prioritizing and making the best use of limited resources. The infant’s condition, birth weight, and gestational age all influenced the care and medications that the NNM infant received. ‘We provide care and treatment based on the infant’s birth weight, gestational age, and risk level.‘ (Participant no. 21).

### Provision of Kangaroo Mother Care

Implementing early initiation of breastfeeding, hypothermia prevention, and early and intermittent Kangaroo Mother Care (KMC) saves the lives of low birth weight babies, premature infants, and asphyxiated infants, according to the participants of a television show at the Neonatal Intensive Care Unit (NICU). The show focused on breastfeeding, hypothermia prevention, and KMC and how they can assist mothers in providing these essential care practices for high-risk babies. It was believed that these interventions could significantly improve the chances of these infants’ survival.

“The baby was covered in a cotton blanket. The mother provided expressed milk on time and followed KMC guidelines. The baby’s condition improved dramatically in a short period, and we discharged the baby after one month.“ (Participant no. 18).

According to healthcare providers, Kangaroo Mother Care, when instructed by health professionals to the baby’s mother or relatives, saved the lives of premature and abandoned infants in the NICU. A sense of hopelessness was always present when caring for infants whose mothers had abandoned them. These infants had limited opportunities to receive KMC as they lacked access to expressed breast milk, relying solely on formula milk. Consequently, the infants were at risk of infection and had a low chance of survival. Some healthcare providers voluntarily initiated providing KMC to these infants in addition to their routine duties to save the infants’ lives.

“The mother left the hospital because her baby had a low chance of survival. We (volunteer healthcare providers) began KMC during our rounds. For such a baby, 24-hour Kangaroo Mother Care was preferable, but it was intermittent due to her mother’s unavailability. I learned how important KMC is for such a baby. Kangaroo Mother Care is the foundation for all critically ill infants, and this baby survived due to volunteering healthcare professionals.“ (Participant no. 6).

The television show about Kangaroo Mother Care in the NICU assists mothers who wish to avoid blame from other mothers due to their baby’s failure to thrive. The privacy and comfort in the NICU also encourage mothers to provide continuous KMC to their infants.

“I remember a female baby weighing 900 grams at 28 weeks gestation. The baby was wrapped in a cotton blanket. The baby’s mother gave her special attention, changing the baby’s clothes daily, expressing breast milk, and implementing KMC.“ (Participant no. 21).

Participants stated that intermittent Kangaroo Mother Care, even while the infant is in the incubator, has the potential to save the lives of very low birth weight infants and improve vital signs. Even if the time was limited, expressed breastfeeding outside of the incubator provided an opportunity to practice KMC. Intermittent KMC for NNM infants had shown to increase mother-infant bonding and resulted in the early physiological and vital sign stabilization of the infants. Preterm and very low birth weight infants are vulnerable to hypoglycemia due to a lack of metabolic reserves and the inability to generate new glucose, according to the participants. Kangaroo Mother Care can assist in thermoregulation and energy conservation by maintaining core temperature and accelerating metabolic adaptation. KMC balanced the sense of hopelessness when caring for NNM babies.

“I recall a 900-gram, 35-week-old male baby with hypoglycemia and abnormal vital signs. It was wrapped in cotton, vital signs were taken every two hours, and it was placed in an incubator. The mother correctly provided intermittent KMC, which contributed to her baby’s survival.“ (Participant no. 13).

The mother’s eagerness to support the medical team by implementing KMC contributed to the infant’s condition improvement. The mother was informed about the infant’s situation, the type of care and treatment that would be provided, and the expected maternal activities. This information helped the mother understand the baby’s situation, and she communicated easily with the medical team. The communication with the medical team enhanced her motivation to provide proper intermittent KMC and express breast milk for the baby’s feeding. The mother supported the medical team by controlling the baby’s movement while receiving oxygen therapy. If the mother noticed anything out of the normal scope regarding the infant, she would alert the medical team. Such cooperation might help her infant survive and avert the sense of hopelessness expressed by healthcare providers when caring for NNM infants.

“There was a very low birth weight and premature baby with apnea. Its vital signs were taken every six hours. The mother was informed of the baby’s condition, and the mother offered her assistance to the medical team. She gave him (the baby) expressed breast milk and correctly applied KMC.“ (Participant no. 7).

### Quick action required at birth

Quick actions by healthcare providers at birth, such as shortening the 2nd stage of labor with vacuum extraction, meconium removal after birth, and immediate resuscitation, have been shown to save neonatal lives, according to the participants. When conditions become life-threatening, responding quickly and implementing necessary interventions can save the lives of high-risk infants.

“I had a fetus with grade II meconium-stained amniotic fluid in the second stage of labor. We used vacuum assistance to speed up the labor. The newborn had difficulty breathing, bluish discoloration, and did not cry as a result of the situation. The resuscitation equipment, including a syringe, bag, and mask, had all been prepared. I quickly cut the cord, wrapped the newborn in a towel, and positioned it close to the radiant warmer. I removed the meconium and resuscitated it for 20 minutes. I believed that such actions would assist the infant’s survival.“ (Participant no. 4).

Teamwork among healthcare providers for prompt action at birth helped newborns survive and averted the sense of hopelessness felt by healthcare providers caring for NNM infants. More collaboration and teamwork are required when attending to a woman in labor with a NNM fetus expected because there would be activities and events needed in such a short period that everyone’s responsibility must be crystal clear. Every member of the team, as well as all the necessary materials, should be ready, the participants stated. Early in the 2nd stage of labor, NICU healthcare providers should be informed about the infant’s life-threatening situation so that they can prepare and come to the rescue.

“I recall a fetus with bradycardia and low saturation in the prolonged second stage of labor. We attempted to reduce labor time by using vacuum extraction, but it was unsuccessful. We used low forceps to expedite labor, but the newborn was not breathing when he was born. We provided bag and mask resuscitation as a team. The infant was then taken to the NICU while I was still dressed in my delivery gown. Through our team efforts, we were able to save such a wonderful baby.“ (Participant no. 25).

Neonatal lives can be saved through admission to a neonatal intensive care unit within the golden hour, according to the participants. Preterm and premature infants must transition from fetal to neonatal life within minutes to hours of birth, but the risks for these NNM infants are high. It is vital to provide appropriate care to these infants in a timely and organized manner, including resuscitation measures and early admission to the neonatal intensive care unit.

“I had one experience with a baby who had very low birth weight, an early gestational age, cleft lip, and respiratory problems. I positioned the baby near the radiant warmer and resuscitated it with a bag and mask for 18 minutes. We began oxygen administration and transferred the infant to the NICU.“ (Participant no. 3).

When putting the sense of hopelessness aside and providing one’s responsibilities in an organized way, labor acceleration, timely meconium removal from asphyxiated infants, prerequisites for resuscitation, and oxygen administration, and then continuous positive airway pressure can save the lives of premature and asphyxiated infants.

“I recall a premature, grade III meconium-stained amniotic fluid and birth asphyxia newborn found in the second stage of labor. Labor was accelerated by vacuum extraction, and the secretion was removed with a valve sucker as soon as the head was delivered. It was dried and wrapped in a towel. We resuscitated it for more than 10 minutes. We gave the baby continuous positive airway pressure, which contributed to its survival, and then referred it to the NICU.“ (Participant no. 18).

## Discussion

Determinants related to caring influence the survival of neonatal near-miss babies in clinical management and care, and these aspects have been described in this qualitative study with healthcare providers in Ethiopia. Healthcare providers perceived a sense of hopelessness when caring for the NNM infant, particularly when providing KMC and taking quick actions when required at birth to save the life of the infant. Healthcare providers working in public hospitals in Ethiopia expressed that a feeling of hopelessness, in combination with unclear personal responsibilities, may have posed a barrier to saving the lives of NNM infants and discharging their mission to provide quick actions and KMC.

The findings of this study shed light on the challenges healthcare providers face when caring for neonatal near-miss (NNM) infants and their impact on the quality of care provided. One notable challenge identified in this research is the tendency of healthcare providers to prioritize infants with better chances of survival when faced with limited resources. This prioritization is based on the belief that NNM infants have a lower likelihood of survival, which can lead to compromised care quality, ultimately increasing the risk of death. This observation aligns with the findings of a cohort study conducted in Brazil, which revealed compromised care quality for NNM infants and its association with higher mortality rates [6].

Additionally, the study highlights the potential consequences of healthcare providers neglecting NNM infants, particularly in cases where responsibilities are unclear or poorly defined. In such situations, NNM infants may not receive timely access to essential care elements, including breastfeeding, maternal care, clinical treatment, and attentive healthcare provider support. This delay in meeting their physiological needs may lead to prolonged instability and increase the risk of infections and other complications. This observation is consistent with the findings of a systematic review [8], which emphasize that patient neglect in healthcare institutions represents a form of disrespect toward women, potentially resulting in inadequate care for their newborns. This underscores the importance of establishing clear and well-defined roles and responsibilities for healthcare providers in the clinical management and care of NNM infants. Furthermore, this study reveals that healthcare providers sometimes use ultrasound to estimate fetal weight during labor. When providers believe that the infant’s chances of survival are low, they may leave the laboring woman unattended during labor, contrary to the recommended standard of care that emphasizes equal care for every woman and baby. This practice puts the infant at risk of hypothermia and infection. It is essential to recognize that such neglect may stem from a lack of up-to-date in-service training on the survival chances of NNM infants, inadequate teamwork, and insufficient training in acute complication management and lifesaving skills. By equipping healthcare providers with the necessary knowledge and skills, healthcare systems can improve the quality of care provided to NNM infants and enhance their chances of survival, aligning with the overarching goal of providing equal care to every woman and child [22]. It is crucial to prioritize training and regular mentoring for healthcare providers, with a specific focus on attitudes and skills related to attending births where quick actions are needed to save the infant’s life. This additional training can help shift healthcare providers’ attitudes away from a sense of hopelessness when caring for NNM infants and toward more effective clinical management and care practices.

This study brings to light several critical issues in the care of neonatal near-miss (NNM) infants, particularly the communication and decision-making processes between healthcare providers and parents. One significant finding is that healthcare providers often inform parents of NNM infants with very low birth weights about the low chances of survival, which may lead to a limitation in care. This approach is consistent with previous research, highlighting the potential consequences of unclear responsibilities in healthcare settings, including a lack of care, dignity, and respect for patients [8]. The study also underscores the importance of understanding that parents may not accept compromises in care and treatment for their NNM infants, regardless of the grim survival rate. Instead, parents may advocate for more attention and care from healthcare providers. The attitudes of healthcare providers toward clinical management and care of NNM infants are shown to have significant implications. These attitudes may put infants at risk for life-threatening complications and can also add emotional burden to parents. Some healthcare providers define NNM infants as those who were on the brink of death but survived due to clinical care, reinforcing the crucial role of healthcare providers in these infants’ survival [23]. The study participants express hope that the concept of NNM would become more widely known among healthcare providers, leading to improved care and treatment for high-risk infants. This knowledge, they believe, could aid in the organized prioritization of care and treatment, especially when dealing with resource limitations. Furthermore, the study underscores the importance of the near-miss classification system [1–3] as a tool for long-term monitoring and follow-up of high-risk infants, aligning with recommendations from previous research [6, 23]. This classification system can serve as a reminder to healthcare providers to maintain vigilance and provide ongoing care to these infants, ultimately enhancing the quality of care provided and healthcare providers’ performance [24, 25]. Focusing on high-risk infants reflects a shift from failure to success in clinical care, contributing to improved care and better outcomes for these vulnerable infants and aligning with the goal of providing quality care to all women and children [22].

The study also highlights specific care practices that are crucial for the survival and well-being of NNM infants. Early initiation of breastfeeding, hypothermia prevention, and early and intermittent kangaroo mother care (KMC) emerge as essential interventions [9–17]. These practices are consistent with existing recommendations, emphasizing the importance of initiating breastfeeding within the first hour of birth to protect against infection and other complications [9]. Similarly, early and intermittent KMC is recognized as a vital component of care for low birth weight, premature, and asphyxiated infants, supported by a multicounty study conducted in low-resource hospitals [14]. The findings of this study align with a broader framework for quality care, emphasizing the role of midwives and healthcare providers in promoting breastfeeding and reducing morbidity [11, 22].

The study’s findings emphasize the significant impact of Kangaroo Mother Care (KMC) provided by health professionals in saving the lives of premature and abandoned infants in the Neonatal Intensive Care Unit (NICU). This aligns with a systematic review study, which underscores KMC’s potential to prevent or reduce morbidities in very low birth weight infants [26]. However, it’s crucial to recognize that awareness, or the lack thereof, plays a significant role in the resistance to KMC practices among individuals or communities. The study highlights the limited opportunities for infants abandoned by their mothers to receive KMC, placing them at risk of infection and reducing their chances of survival. It’s worth noting that some healthcare providers take the initiative to provide KMC to abandoned infants, even beyond their routine duties, underscoring their commitment to saving infants’ lives.

The study’s findings regarding intermittent KMC, even while infants are in incubators, are supported by a meta-analysis of randomized controlled trials, which suggests that KMC in the NICU is safe and has a positive effect on some physiological parameters of preterm infants [16]. Intermittent KMC is shown to enhance mother-infant bonding and early physiological and vital sign stabilization. Furthermore, the study suggests that KMC can be practiced during expressed breastfeeding sessions outside the incubator, contributing to bonding, psychological well-being, and milk production. This aligns with the consensus that intermittent KMC is a feasible approach for increasing the likelihood of infants benefiting from exclusive breastfeeding and weight gain [10, 15].

The study also highlights the crucial role of mothers’ eagerness to support the medical team and implement KMC, which positively influences infants’ conditions. This aligns with previous research indicating that mothers become motivated when they realize their role in assisting preterm infants through KMC [17]. Adequate KMC counseling is identified as an essential component for long-term acceptance and uptake, emphasizing the importance of clear communication between healthcare providers and mothers.

The study underscores the significance of midwives’ prompt actions during childbirth, such as vacuum extraction to shorten the second stage of labor, meconium removal, and immediate resuscitation, which can save neonatal lives. This aligns with the midwifery and quality care framework and is supported by research from Jordan and Nepal [21, 22, 27, 28]. Additionally, the study’s findings highlight the impact of midwives’ experience on their decision-making, further reinforcing the role of skilled midwives in saving both mothers and newborns [29, 30]. Collaboration and timely organized interventions are essential when attending to women in labor, given the potential for rapid developments and critical events during childbirth.

Finally, the study emphasizes the effectiveness of continuous positive airway pressure and timely admission to a neonatal intensive care unit (NICU) within the golden hour in saving lives. These interventions align with established evidence-based practices that contribute to improved long-term health outcomes [30, 31]. It is noted that these interventions are part of a comprehensive care package for high-risk newborns, emphasizing the importance of providing timely and organized care, especially for preterm and premature infants who transition from fetal to neonatal life within minutes to hours of birth. Ethiopia’s commitment to implementing these care packages to achieve the Sustainable Development Goal of reducing neonatal mortality to 12 per 1000 live births is commendable [5].

### Strengths and limitations

The study’s strengths lie in its diverse participant characteristics, depth of exploration, robust data collection methods, pilot testing, and trustworthiness strategies. The study collected data from healthcare providers of varying ages, backgrounds, and clinical experience in labor and NICU wards. This diversity enhances the richness and comprehensiveness of the study’s findings, reflecting a variety of perspectives and experiences among participants [32]. The study prioritized in-depth exploration of participants’ thoughts, experiences, and viewpoints. The difference in educational and work experiences between midwives, nurses and pediatricians included in the study strengthen the trustworthiness and transferability of the findings [32]. The study captured the depth and richness of their experiences. This approach allowed for a comprehensive understanding of the phenomenon under investigation, as the content of sub-categories was abstracted into a main category. The use of interviews as the data collection method enabled researchers to gather rich and detailed data through one-on-one conversations with participants. This approach facilitated a deeper exploration of participants’ perspectives, contributing to the comprehensiveness and nuance of the information gathered. The interview guide was pilot tested, and an Amharic version was created with minimal prompting. This ensured that healthcare providers could express their experiences and perceptions in their own words, contributing to the authenticity of the data. Several trustworthiness strategies were implemented, following criteria from Elo and Kyngas [18]. This included participant selection to achieve credibility, audio recording and verbatim transcription of interviews, rigorous translation processes, and the involvement of all authors in the analysis process. Discussions among authors during the analysis phase helped minimize bias and ensure the accuracy of results. However, limitations include a small sample size and context-specific results. The study’s sample size is relatively small, which can limit the transferability of findings. It may be challenging to apply the study’s results to different contexts or settings, reducing the generalizability of the findings. Researchers’ personal beliefs and perspectives were held back and reflected upon to not influence the analysis and interpretation of the data. While efforts were made to minimize any bias, it is challenging to completely eliminate subjectivity in qualitative research. The study adopted an inductive approach [32], deriving transferability based on patterns observed in the data. This approach requires specific analysis skills, interview techniques, and a willingness to engage in open dialogue with participants. Qualitative research, by its nature, provides context-specific insights. While these insights are valuable for understanding complex phenomena, they may not be easily applicable or transferable to different contexts or populations. Qualitative research, despite its limitations, offers valuable insights that complement the quantitative research data provided in three previous studies [1–3] in an ongoing research project with several studies included. The qualitative results are essential to gain a better understanding of determinants contributing to the survival of neonatal near-miss babies and to identify any barriers encountered, as reported by the experiences of healthcare providers in public hospitals of Amhara Regional State, northwest Ethiopia.

### Implications to clinical practice

The findings underscore the critical need for enhanced training and mentoring initiatives aimed at equipping healthcare providers with the knowledge and skills required to fulfill their mission of delivering appropriate care and management to NNM infants. Specifically, addressing the issue of unclear responsibilities and providing guidance on how to effectively implement KMC and facilitate breastfeeding can play a pivotal role in improving the outcomes of these vulnerable infants. Encouraging a more optimistic outlook can foster a proactive approach to caregiving and empower healthcare providers and parents alike to work together in providing the best possible care and management for NNM infants. This study emphasizes the importance of comprehensive interventions that address training, attitudes, and clear responsibilities to improve the care and survival prospects of these infants. This underscores the significance of continuous mentoring and training in the domains of clinical management, care, and the utilization of the NNM assessment scale, particularly for healthcare providers dealing with near-miss infants who may initially feel hopeless. Greater emphasis should be placed on raising awareness among healthcare providers about the prevention of hypothermia, skin-to-skin care, KMC, and the importance of expressing breast and neonatal resuscitation.

## Conclusion

In conclusion, this study sheds light on the significant challenges healthcare providers face when caring for neonatal near-miss (NNM) infants. The presence of a pervasive sense of hopelessness and unclear responsibilities within the healthcare system appears to be a significant impediment to the effective provision of timely interventions, such as quick actions at birth and Kangaroo Mother Care (KMC), that can save the lives of NNM infants. In essence, this study emphasizes the importance of comprehensive interventions that address training, attitudes, and clear responsibilities to improve the care and survival prospects of NNM infants.

## Data Availability

This manuscript contains all the data generated or analyzed during the study. We do not intend to share the data due to the presence of participants’ identifications, including names, in the raw data, as it is a qualitative study. However, data can be made available from the corresponding author upon reasonable request.

## Abbreviations

IRB: Institutional Review Board
KMC: Kangaroo Mother Care
NICU: Neonatal Intensive Care Unit
NNM: Neonatal Near Miss
SDG: Sustainable Development Goal
